# Bacteria‐Elicited Specific Thrombosis Utilizing Acid‐Induced Cytolysin A Expression to Enable Potent Tumor Therapy

**DOI:** 10.1002/advs.202105086

**Published:** 2022-04-11

**Authors:** Wenjun Qin, Wenxuan Xu, Longyu Wang, Debao Ren, Yibin Cheng, Wen Song, Tao Jiang, Lixin Ma, Cheng Zhang

**Affiliations:** ^1^ Ministry of Education Key Laboratory for the Green Preparation and Application of Functional Materials School of Materials Science and Engineering Hubei University Wuhan 430062 P. R. China; ^2^ State Key Laboratory of Biocatalysis and Enzyme Engineering Hubei Key Laboratory of Industrial Biotechnology School of Life Sciences Hubei University Wuhan 430062 P. R. China; ^3^ Institute of Biology and Medicine & College of Life Science and Health Wuhan University of Science and Technology Wuhan 430081 P. R. China

**Keywords:** acid‐induced, bacteria, nutrient interruption, thrombosis, tumor therapy

## Abstract

Given the special microenvironment of solid tumors, live microorganisms have emerged as drug delivery vehicles and therapeutic agents. Here, an acid‐induced therapeutic platform is constructed using attenuated *Escherichia coli* to express the cytolysin A protein. The bacteria can target and colonize tumor tissues without causing notable host toxicity. Bacterial infection can disrupt blood vessels and trigger thrombosis in tumor tissues, resulting in the cut‐off of nutrient supply to tumor cells and the arrest of tumor growth. The expression of cytolysin A induced by the acidic tumor microenvironment further strengthens thrombosis and provides a complementary therapeutic option due to its pore‐forming function. In a xenograft mouse tumor model, this strategy reduces tumor proliferation by 79% and significantly prevents tumor metastasis, thus paving a new avenue for bacteria‐based tumor therapy.

## Graphical abstract

A bacteria‐based platform is constructed to inhibit tumor growth and metastasis. Selective colonization of bacteria in tumor tissues causes thrombosis, thereby blocking the tumor nutrients supply. Cytolysin A expressed under the inductive influence of the tumor acid microenvironment strengthens this therapeutic effect.

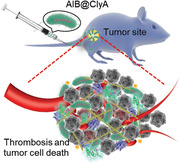

## 1 Introduction

Malignant tumors affect the quality of life and health of many people worldwide.^[^ ^1^ ^]^ To eliminate the threat posed by cancer, conventional therapeutic regimes such as radiotherapy, surgery, and chemotherapy based on various mechanisms are in clinical practice.^[^ ^2^ ^]^ Although these regimens inhibit tumor growth to some extent, multiple treatments are required during the rehabilitation period, sometimes resulting in severe side effects including therapeutic resistance and causing anxiety.^[^ ^3^ ^]^ Further, the emergence of metastasis and its recurrence greatly limit the extensive and in‐depth application of conventional therapeutic regimens.^[^ ^4^ ^]^ Due to rapid developments in the fields of nanotechnology and biomedicine, precision therapies based on targeting tumors are highly sought after.^[^ ^5^, ^6^, ^7^ ^]^

The complex vascular network of solid tumor tissues is an important feature.^[^ ^8^ ^]^ The vasculature is formed during tumorigenesis and development, which transports nutrients and oxygen to tumor tissues and clears the metabolic waste and carbon dioxide that is generated.^[^ ^9^ ^]^ Furthermore, the blood vessels also provide convenient access for tumor cells for distal metastasis.^[^ ^10^ ^]^ Therefore, attention has focused on targeting tumor blood vessels to suspend tumor progression by disrupting the function of the vasculature.^[^ ^11^, ^12^ ^]^ Based on the different mechanisms involved, two vessel targeting therapies are prevalent, that is, antiangiogenesis and destroying or obstructing the established blood vessels. For antiangiogenesis, the angiogenesis pathways are disrupted by vascular endothelial growth factor antibodies or using copper ion inhibitors to prevent the formation of new blood vessels.^[^ ^13^, ^14^ ^]^ The destroying or obstructing established blood vessels strategy is employed using vascular disrupting or embolic agents.^[^ ^15^, ^16^ ^]^

Notably, vascular occlusion can deprive tumors of oxygen and nutrients within hours after thrombosis and decrease the risk of resistance development.^[^ ^17^ ^]^ As blood vessels of solid tumors are essentially similar, thrombosis‐based vessel targeting therapy can be widely applied to target many types of tumors.^[^ ^18^ ^]^ However, because nonselective thrombosis may induce coagulation events and cause severe side effects in healthy tissues, it is critical to precisely control thrombosis in tumor tissues.^[^ ^19^, ^20^ ^]^ Further, single thrombosis‐based therapy is usually too weak to eliminate the tumor vasculature, as the escaped and newly formed blood vessels coexist and attempt to reestablish the oxygen and nutrient supply.^[^ ^21^ ^]^ Therefore, there is an urgent need to design a comprehensive therapeutic strategy that can simultaneously induce thrombosis in tumor areas and function as a complementary therapy to strengthen curative effects.

**Scheme 1:**
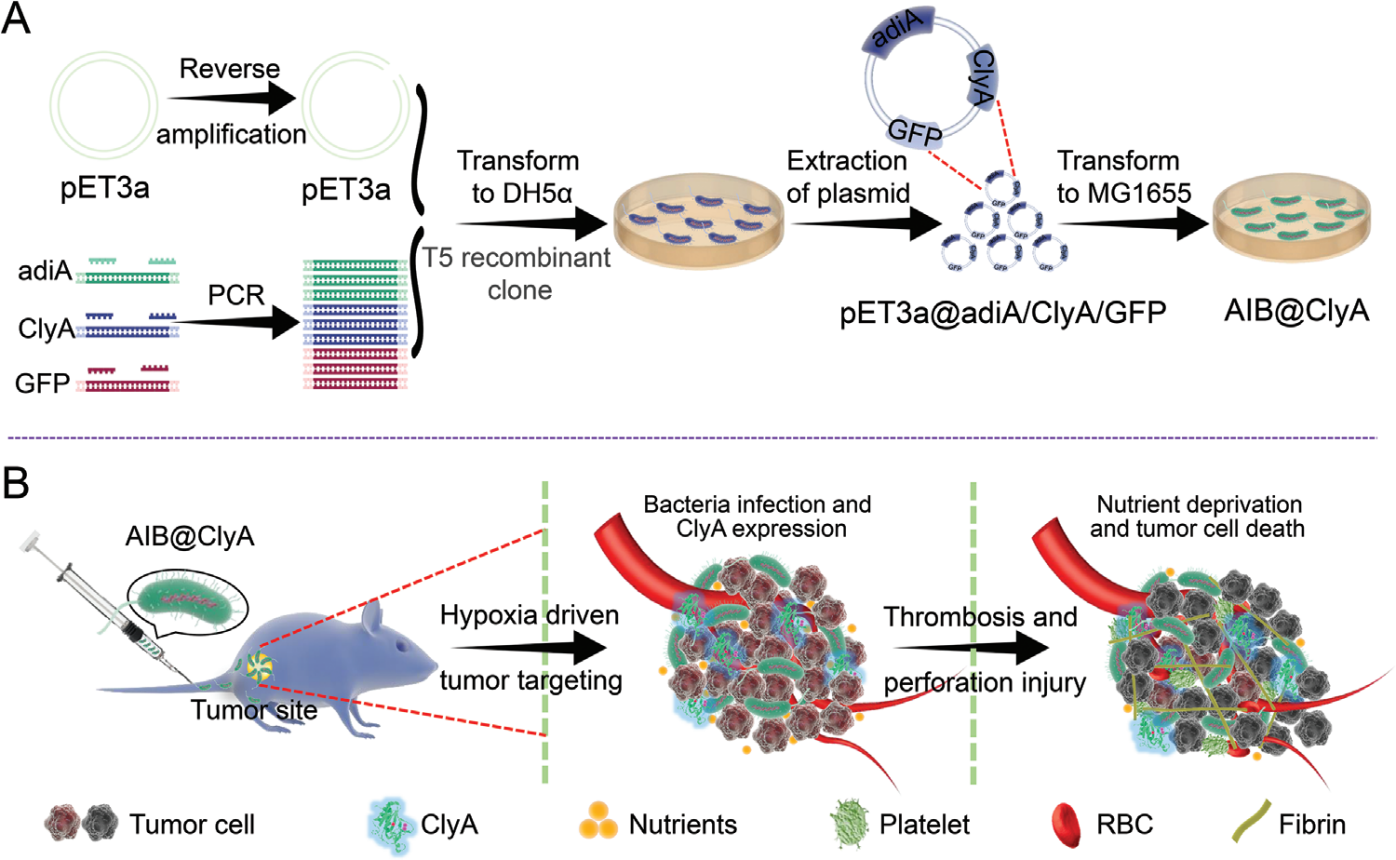
Preparation and in vivo antitumor therapy with AIB@ClyA. A) Schematic diagram of the preparation process of acid‐induced ClyA expression in engineered *E.coli*. B) Schematic illustration of bacteria‐triggered tumor thrombosis and the subsequent nutrient deprivation for tumor ablation in vivo.

At the end of the 19th century, surgeon William Coley accidentally observed tumor ablation after injecting heat‐killed streptococcal organisms in conjunction with *Serratia marcescens* into sarcoma patients, opening the door for bacteria‐based tumor therapies to develop.^[^ ^22^ ^]^ Because of specific characteristics, bacteria have natural advantages in the field of tumor therapy. For instance, facultative anaerobes and obligate anaerobes selectively home to and colonize solid tumor tissues as these have hypoxic and immunosuppressive microenvironments, making these bacteria potential drug delivery carriers.^[^ ^23^, ^24^ ^]^ However, the early treatment strategies that have been developed are complex in design and have poor efficacy. With the development of advances in synthetic biology, bacteria‐based tumor therapy is now viewed as being efficient and diverse.^[^ ^25^ ^]^ By transfecting customized plasmids into bacteria after genetic engineering, bacteria can express specific proteins in tumor tissues that can kill tumor cells, thus playing the role of therapeutic agents.^[^ ^26^, ^27^ ^]^ Importantly, bacterial infection in tumor tissues may result in the release of pro‐inflammatory factors and disrupt the tumor vasculature, causing platelet aggregation because of platelet activation and fibrin formation within tumor tissues where coagulation may occur.^[^ ^28^, ^29^ ^]^ Given the versatility of bacteria, bacteria‐based therapeutic platforms can be potentially used for the development of effective multimodal synergistic therapies against malignant tumors. Herein, we established a therapeutic platform (AIB@ClyA) for acid‐induced bacterial expression of cytolysin A (ClyA) protein by employing the nonpathogenic *Escherichia coli* (*E. coli*) strain K‐12 (MG1655). As illustrated in **Scheme** **1**A, custom‐designed plasmids (pET3a) with an acid‐sensitive promoter (adiA) and the green fluorescent protein (GFP)‐conjugated cytolysin A (ClyA) gene were transformed into MG1655. The AIB@ClyA showed preferential tumor‐targeting capacity and sensitivity to acid responsiveness. After intravenous injection, the AIB@ClyA modality restrained tumor growth via nutrient exchange blockage and membrane perforation injury and impeded tumor metastasis by cutting off the metastatic process (Scheme 1B). We believe that the AIB@ClyA strategy can potentially tackle the major challenges that arise during tumor therapy and can expand the current applications of bacteria‐based platforms for precision therapy.

## 2 Results and Discussion

### 2.1 Construction and pH Sensitivity Evaluation of AIB@ClyA

The transfer plasmid pET3a@adiA/ClyA/GFP was constructed based on a previously reported protocol with minor modifications (Table S1, Supporting Information).^[^ ^30^ ^]^ We recombinantly fused the gene fragment for ClyA containing the adiA promoter and GFP tag into the vector plasmid. The ClyA gene was derived from *Salmonella typhimurium*, and the translated protein has the ability of membrane perforations to damage tumor cells.^[^ ^31^ ^]^ Considering the biosafety requirements in vivo, non‐pathogenic *E. coli* MG1655 was selected to reduce the side effects.^[^ ^32^, ^33^ ^]^ Therefore, after mass replication of the transfer plasmid in *E. coli* DH5*α*, the plasmid was extracted and transformed into *E. coli* MG1655 for subsequent experiments.

**Figure 1:**
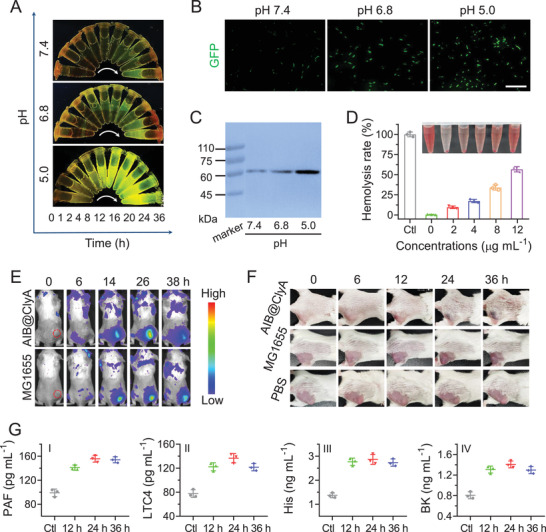
Expression of ClyA and thrombus induction in vivo. A) Time‐dependent GFP expression at various pH values. B) GFP expression at the 16 h culture time point observed by inverted fluorescence microscope. Scale bar: 20 µm. C) Identification of GFP‐tagged ClyA expression via Western blot assay. D) Hemolysis‐based quantification of erythrocytes at various concentrations of GFP‐tagged ClyA. Insert: Picture of hemolysis after centrifugation. E) Time‐dependent fluorescence images of CT26 tumor‐bearing mice injected with DiR labeled bacteria. F) Photographs of BALB/c mice bearing CT26 tumors before or after injection of bacteria with doses of 10^8^ CFU. G) The levels of inflammatory factors in sera from CT26 tumor‐bearing mice isolated at different time points after AIB@ClyA injection. Data are presented as the means ± SD.

Before verifying the acid responsiveness of AIB@ClyA, we investigated the changes in bacterial viability after plasmid transformation. Compared with MG1655, the spread plate assay showed that numerous AIB@ClyA still survived even in weak acid environments (Figure S1, Supporting Information). The expression of ClyA in different pH environments was visually confirmed via analysis of the GFP fluorescence intensity. From the results shown in **Figure** **1**A, we observed an increase in ClyA expression fluorescence intensity with the extension of culture time. In marked contrast, the incremental changes in GFP expression observed in pH 5.0 cultures were significantly faster than at pH 7.4 and pH 6.8. A similar result was observed using an inverted fluorescence microscope (Figure 1B). After culturing for 16 h, a greater number of bacteria were involved in the expression of GFP, and GFP expression levels in pH 5.0 medium were increased. As a control, MG1655 used alone did not express GFP in the same conditions (Figure S2, Supporting Information). To analyze the expression of ClyA, the protein was obtained after multiple centrifugations and filtration steps for Western blot analysis. As shown in Figure 1C, the protein band indicating GFP‐tagged ClyA was consistent with the theoretical value of 61 kDa, and the protein amounts increased with a decrease in the pH value of the culture medium (Figure S3, Supporting Information). Therefore, according to our design, the expression of ClyA could be accurately manipulated by changing the ambient pH. Next, the membrane perforation caused by ClyA was evaluated via a hemolysis assay. Erythrocytes incubated with pure water were used as a control, and the culture medium for other groups included phosphate buffer solution (PBS). As shown in Figure 1D, with increasing ClyA concentration in the buffer solution, hemolysis showed a corresponding increase. When the protein concentration incubated with the erythrocytes was 12 µg mL^−1^, the hemolysis rate calculated by UV–vis absorbance reached ≈57%. The excellent membrane perforation ability of ClyA caused significant cytotoxicity (Figure S4, Supporting Information), which has been shown in our previous work.^[^ ^30^ ^]^

### 2.2 Tumor Vascular Disruption and Thrombosis In Vivo

Before exploring thrombosis in tumor tissues, a mouse model bearing CT26 colorectal tumor was established to investigate the bacterial bio‐distribution in vivo. Mice with tumor volumes of ≈100 mm^3^ were intravenously injected with 1,1‐dioctadecyl‐3,3,3,3‐tetramethyl indotricarbocyaine iodide (DiR)‐labeled bacteria. After intravenous injection, small animal imaging system results showed that both MG1655 and AIB@ClyA were enriched in tumor tissues with time (Figure 1E). About 26 h after injection, the enrichment of bacteria in tumor sites reached maximal levels (Figure S5, Supporting Information). Similar results were observed using fluorescence imaging of isolated main organs and tumor tissues (Figure S6, Supporting Information). Tumor targeting using bacteria may induce thrombosis at tumor tissues; therefore, we evaluated intratumoral thrombosis by intravenous injection of MG1655 or AIB@ClyA into CT26 tumor‐bearing mice, and an injection dose of 1×10^8^ colony‐forming units (CFU) per mouse was used; an equal volume of PBS was used as the control. With the enrichment and proliferation of bacteria in tumor tissues over time, we observed that the color of the tumor sites gradually darkened (Figure 1F). The darkening of the tissue with AIB@ClyA was stronger than that for the group injected with MG1655, and this may be due to the acid‐induced ClyA expression in tumor tissues. The degree of darkening was closely related to the dose of injected bacteria (Figure S7, Supporting Information). In sharp contrast, there was almost no color change of tumor sites in the PBS‐injected group. We inferred that this difference could be attributed to intratumoral thrombosis, which was caused by the concentrated blood coagulates within tumor tissues.^[^ ^28^ ^]^

**Figure 2:**
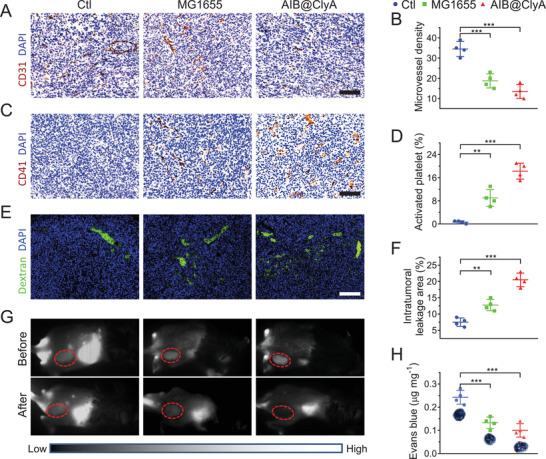
Thrombosis at tumor sites. A) Images of tumor vessels in different treatment groups by CD31 labeling. Scale bar: 100 µm. B) Microvessel density of tumor sections evaluated by calculating the number of CD31**^+^** (brown) in four fields per group. C) Images of activated platelets to detect thrombosis in different treatment groups by CD41 labeling. Scale bar: 100 µm. D) Activated platelets in tumor sections evaluated by calculating the CD41**^+^** area (brown) in four fields per group. E) The permeability of tumor vessels in different groups monitored by FITC‐dextran. Scale bar: 200 µm. F) Intratumoral leakage area estimated by calculating the area of dextran**^+^** (green) in four fields per group. G) Photographs of intratumoral leakage in different groups visualized by near‐infrared‐II luminescence imaging. H) Evans blue content and the corresponding tumor tissue images in different treatment groups. All data are shown as the means ± SD and statistical analyses were performed by the unpaired two‐tailed Student's *t*‐test. ***p < 0.001, **p < 0.01.

To confirm the above hypothesis, we checked for the presence of vasodilator inflammatory factors including platelet‐activating factor (PAF), leukotriene C4 (LTC4), histamine (His), and bradykinin (BK) in the serum of mice treated with AIB@ClyA. We observed an increase in the level of all the cytokines within 36 h after injection (Figure 1G), which could disrupt the vasculature and lead to the blood influx and platelet aggregation in the extravascular area.^[^ ^34^ ^]^ Furthermore, tumor tissues were collected at 36 h post‐injection for immunohistochemistry and immunofluorescent staining. CD31 and CD41, typical markers of vascular endothelial cells and platelets, respectively, were used for staining tumor sections to visualize the tumor vessels and platelet aggregation. Confocal microscopy analysis showed that tumor sections from the control (Ctl) group displayed strong CD31 fluorescence and almost no CD41 fluorescence (**Figure** **2**A,C), indicating the presence of an abundant vascular network in tumor tissues without platelet activation. After being treated with MG1655 and AIB@ClyA, the fluorescence signal of CD31 and CD41 gradually decreased and increased, respectively. Quantification of the CD31**^+^** number and CD41**^+^** area was performed, and the results from Figure 2B,D show that the AIB@ClyA‐treated group exhibited a 21% decrease in intratumoral microvessel density and a 17.6% increase in platelet aggregation. Additionally, we evaluated the hemoglobin content at tumor sites via the UV–vis absorption analysis of hemoglobin at different time points. Our results showed that the hemoglobin content in tumor tissues was increased within 24 h after injection of AIB@ClyA (Figure S8, Supporting Information). Therefore, bacterial colonization and ClyA expression within tumor sites can trigger intratumoral thrombosis by disrupting tumor vessels.

Vascular collapse can cause increased vascular permeability and reduced vascular perfusion.^[^ ^35^ ^]^ Using FITC‐labeled dextran, we evaluated the permeability of tumor vessels by measuring vascular leakage. At 30 min post‐injection of FITC‐labeled dextran, tumor tissues from different groups were harvested and sliced for confocal imaging (Figure 2E). Compared with the control group, the dextran leakage area in tumor sections from MG1655 and AIB@ClyA‐treated mice was increased to varying levels. Statistical analysis revealed that the dextran leakage area in AIB@ClyA‐treated mice had increased by 7.8% relative to MG1655‐treated mice (Figure 2F), indicating a further disruption of tumor vessels due to AIB@ClyA treatment. Similarly, vascular perfusion was evaluated through intravenous injection of Evans blue, a macromolecular dye for blood volume determination. At 3 h post‐injection with Evans blue, tumor tissues from different groups were harvested for photographic analysis and soaked in formamide to extract Evans blue. From the images in Figure 2H, we can deduce that the color depth of Evans blue in the control group was much stronger than that in the MG1655 and AIB@ClyA groups. The standard curve analysis of concentration as a function of absorption intensity (Figure S9, Supporting Information) showed that AIB@ClyA treatment caused a 58.7% drop in the retention of Evans blue in tumor tissues, indicating the deterioration of tumor vascular perfusion. To evaluate intratumoral thrombosis, the accumulation of indocyanine green (ICG) at tumor sites was visualized using the NIR‐II luminescence imaging system before and after various treatments. As shown in Figure 2G, the accumulation of ICG at the tumor site was almost unchanged in the control group before and after PBS treatment. However, after 48 h of treatment with MG1655 or AIB@ClyA, a decrease in ICG enrichment at tumor sites was observed. In particular, ICG was hardly enriched at the tumor site after treatment with AIB@ClyA. These results indicate that the AIB@ClyA strategy could effectively disrupt tumor vessels to increase vessel permeability and reduce vascular perfusion, leading to thrombosis.

### 2.3 Nutrient Deprivation Analysis

**Figure 3:**
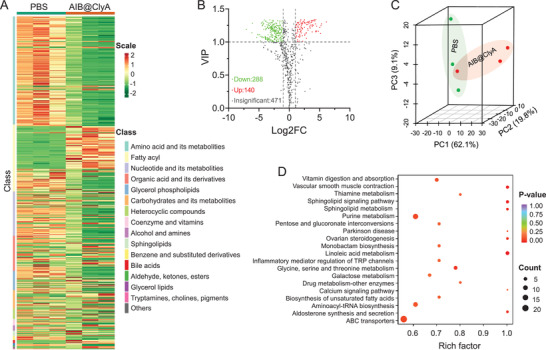
Nutrient deprivation analysis. A) Heat map and cluster analysis of intracellular substances and metabolites in CT26 tumor‐bearing mice after treatment with PBS and AIB@ClyA. B) Volcano plot comparing substances and metabolites between PBS and AIB@ClyA treated nice. C) PCA scores of the substances and metabolites with significant differences between the indicated groups. D) KEGG enrichment analysis of metabolites between PBS and AIB@ClyA treatment groups.

Tumor vessels play an important role in nutrient supply, and intratumoral thrombosis may cause dysfunction of substance exchange and result in abnormal metabolism. Herein, metabonomic analysis was conducted on CT26 tumor‐bearing mice to evaluate tumor metabolism by gas chromatography‐mass spectrometry (GC‐MS).^[^ ^36^ ^]^ As shown in **Figure** **3**A, 899 intracellular substances and metabolites were identified, which were classified into two unsupervised horizontal clusters via cluster analysis. After preliminary classification, we found that the AIB@ClyA‐treated group showed down‐regulation of amino acid, nucleotides, and their metabolites, which are important components of cellular activities. Further, after filtering the data using criteria such as > twofold change (FC) or < 0.5‐FC and variable importance in projection (VIP) score > 1, we found that 428 kinds of substances and metabolites showed an abnormal change, including 140 and 288 that were up and down‐regulated, respectively (Figure 3B). Additionally, the top 10 changes in the AIB@ClyA‐treated group related to coenzyme and fatty acyl metabolism (Figure S10, Supporting Information). Principal components analysis (PCA) was utilized to evaluate the variation between substances and metabolites in AIB@ClyA‐treated mice relative to the PBS group, and the results showed significant differences (Figure 3C). We inferred that these abnormal fluctuations were due to the nutrition supply deficiency caused by thrombosis.

Furthermore, we performed a Kyoto Encyclopedia of Genes and Genomes (KEGG) database search to evaluate the change in biological responses and pathways after AIB@ClyA treatment (Figure 3D). The KEGG annotation classification statistical analysis showed that differentially expressed genes were mainly associated with biosynthetic and metabolic processes in the biological process ontology. Signal transduction and molecular transport were also in the list, which indicated that thrombus hindered signal and material exchange between cells. KEGG enrichment results confirmed similar results showing that differentially expressed genes were notably enriched for biosynthetic and metabolic processes, implying that thrombus induced by vascular destruction affected tumor cell growth. The exact pathways involved in organismal systems, metabolism, genetic information processes, environmental information processes, and cellular processes were further analyzed, and the results showed that several branch pathways were affected (Figure S11, Supporting Information).

### 2.4 In Vivo Therapeutic Effect

**Figure 4:**
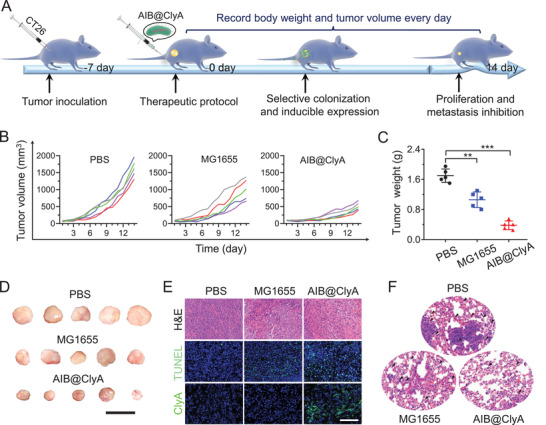
In vivo therapeutic effect. A) Therapeutic schedule of AIB@ClyA administration in a CT26 mouse subcutaneous tumor model. B) Tumor volume profiles of different treated groups in two weeks. C) Tumor weights of mice with different treatments on the 14th day. Data are presented as the means ± SD and statistical analyses were performed by the unpaired two‐tailed Student's *t*‐test. ***p < 0.001, **p < 0.01. D) Photograph of harvested tumor tissues after different treatments on the 14th day. Scale bar: 3 cm. E) H&E staining, TUNEL immunofluorescence staining, and ClyA expression marked by GFP in tumors from mice after different treatments. Scale bar: 200 µm. F) H&E‐stained lung sections from mice after different treatments. The arrows indicate metastasis.

The in vivo therapeutic effect of MG1655 and AIB@ClyA was further evaluated in CT26 tumor‐bearing mice. As per the therapeutic protocol outlined in **Figure** **4**A, the body weight and tumor volume in treated mice were recorded every day during treatment. Compared with the PBS group (Figure 4B,C), mice treated with MG1655 showed tumor inhibition effects. We inferred that this was caused by the formation of microthrombosis after bacterial colonization of tumor tissues, which altered the microenvironment of the tumor cells. Further, a therapeutic efficacy with 79% tumor suppression was observed in the AIB@ClyA‐treated group. These therapeutic effects were confirmed from the exfoliated tumor images on the 14th day (Figure 4D). Next, hematoxylin and eosin (H&E) staining and terminal deoxynucleotidyl transferase‐mediated dUTP‐biotin nick end labeling (TUNEL) staining were used to further investigate tumor suppression in vivo (Figure 4E). The AIB@ClyA‐treated group showed nuclear condensation and cellular apoptosis in H&E and TUNEL staining analysis, respectively, indicating the severe tumor cell damage caused. The results showed a reduced response in the MG1655‐treated group and were mainly caused by the differential expression of ClyA between the two treatment groups (Figure 4E). These results indicate the improved therapeutic efficacy of mice treated with AIB@ClyA. After extending the treatment period to 30 days, mouse lung sections from different groups were stained by H&E to evaluate metastasis (Figure 4F). In marked contrast, the number and area of lung metastases were decreased significantly in the AIB@ClyA‐treated group, confirming the anti‐metastasis ability of AIB@ClyA. Thus, the AIB@ClyA strategy exhibited efficient tumor suppression and anti‐metastasis ability because of the formation of thrombosis and with the assistance of ClyA. Besides, the survival rates on the 30th day indicated that AIB@ClyA treatment could delay the life of tumor‐bearing mice (Figure S12, Supporting Information).

### 2.5 Biosafety Evaluation In Vivo

**Figure 5:**
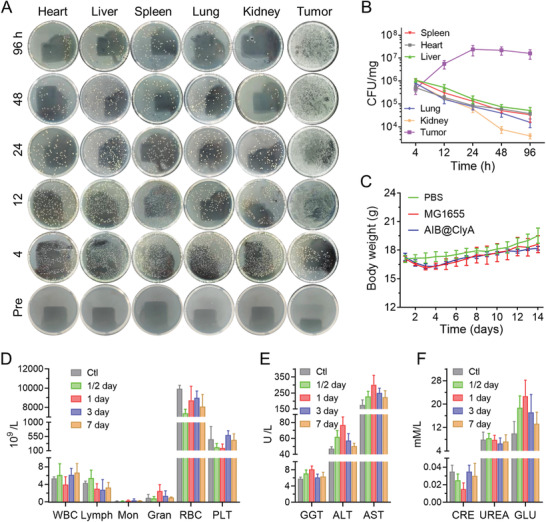
In vivo biosafety. A) Representative photographs of solid LB agar plates, and B) quantification of bacterial colonization in different organs harvested from mice bearing the CT26 tumor before or after bacterial injection. C) Mouse weight change during treatment. D) Blood routine assay at various time points after intravenous injection of AIB@ClyA. E) Liver function‐related enzyme concentration and F) kidney function‐related substrate concentration at various time points after intravenous injection of AIB@ClyA. All data are presented as the means ± SD.

Considering the widespread concerns related to bacteria‐based tumor therapy, we comprehensively assessed the biosafety of the AIB@ClyA strategy using several analyses. Before treatment, the retention of bacteria in CT26 tumor‐bearing mice was monitored via intravenous injection of AIB@ClyA with a dose of 10^8^ CFU per mouse. At 4, 12, 24, 48, and 96 h after injection, the mice were euthanized and their main organs were harvested. After homogenization and serial dilution, the slurry of organs and tumor tissues was plated on Luria–Bertani (LB) plates to count the CFU (**Figure** **5**A). As shown in Figure 5B, bacteria were enriched and colonized in tumor tissues, whereas the bacteria in the main organs were gradually eliminated within 96 h. This phenomenon was attributed to the characteristics of facultative anaerobes and the immune system of organisms. During treatment, the bodyweight of mice from MG1655 and AIB@ClyA‐treated groups did not show significant loss within the normal range in the first three days, and subsequently showed a growing trend (Figure 5C). To evaluate the peripheral blood cells, liver function, and kidney function, blood was collected for blood routine and blood biochemistry assays during treatment. Compared with control mice, the concentration of blood cells including white blood cells (WBC), lymphocytes (Lymph), monocytes (Mon), granulocytes (Gran), red blood cells (RBC), and platelets (PLT) in AIB@ClyA‐treated mice exhibited normal fluctuation (Figure 5D). Liver function‐associated enzymes such as gamma‐glutamyl transpeptidase (GGT), alanine transaminase (ALT), and aspartate aminotransferase (AST) showed a modest increase during the first day (Figure 5E). Simultaneously, two kidney function‐associated biomarkers creatinine (CRE) and glucose (GLU) fluctuated greatly, but were within the normal range (Table S1, Supporting Information), and returned to reference values after a week. Urea, another biomarker related to kidney function, was maintained at a steady level (Figure 5F). In addition, after a two‐week treatment period, the main organs including the heart, liver, spleen, lung, and kidney were collected for H&E staining analysis, and no visible lesions were observed (Figure S13, Supporting Information). These comprehensive examinations verified that AIB@ClyA‐based tumor therapy does not elicit significant host toxicity.

## 3 Conclusions

We have engineered a bacteria‐based platform for acid‐induced ClyA expression. Due to the unique features of facultative anaerobes, the selected *E.coli* strain specifically colonized tumor tissues with little retention in major organs and insignificant side effects in treated mice. Inflammatory factors released by intratumoral bacterial infection disrupted tumor vessels, which in turn triggered tumor thrombosis. After the formation of blood coagulation within tumor tissues, the nutrient supply for tumor cell growth was cut off. The ClyA induced in the acidic tumor microenvironment and caused membrane perforation exacerbated blood coagulation and further damaged tumor cells. In a mouse xenograft model, this strategy arrested primary tumor growth and prevented the formation of metastases. Although bacteria‐based therapies are in the initial development stages, we believe that they have great potential to improve the current tumor therapies.

## 4 Experimental Section

### Materials

1,1‐dioctadecyl‐3,3,3,3‐tetramethyl indotricarbocyanine iodide (DiR) was purchased from Shanghai Yeasen Biological Technology Co., Ltd. (China). Inflammatory factor kits including histamine (His), bradykinin (BK), platelet‐activating factor (PAF), and leukotriene C4 (LC4) were purchased from Shanghai Enzyme‐linked Biotechnology Co., Ltd. (China). The *E. coli* MG1655 and *E. coli* DH5*α* strains were bought from Beijing TransGen Biotech Co., Ltd. (China). The mouse colon carcinoma CT26 cells were provided by China Center for Type Culture Collection. The reagents fetal bovine serum (FBS), Roswell Park Memorial Institute‐1640 medium (RPMI‐1640), PBS, trypsin, and 3‐[4,5‐dimethylthiazol‐2‐yl]‐2,5‐diphenyltetrazolium‐bromide (MTT) were bought from Invitrogen Corp. Cell staining buffer, anti‐CD31, and anti‐CD41 were purchased from Biolegend. TUNEL apoptosis assay kit and FITC‐dextran were obtained from Beyotime Co., Ltd. and Shanghai Aladdin Bio‐Chem Technology Co., Ltd., respectively. All other solvents and reagents were analytically pure and used directly.

### Construction of AIB@ClyA

The ClyA gene from *Salmonella typhimurium* genomic DNA and the DNA for the adiA promoter region were codon‐optimized and synthesized by Wuhan GeneCreate Biological Engineering Co., Ltd. (China). The pET3a plasmid was linearized using BglII and BamHI. The synthesized DNA of the adiA promoter region and ClyA were amplified using associated primers that had a 15 nt homology with the linearized plasmid. The GFP gene was amplified using associated primers that had a 15 nt homology with the ClyA sequence. The ClyA/GFP fragment was produced using overlap PCR. Next, these fragments were cloned into the pET3a vector to construct the acid‐induced expression vector (pET3a@adiA/ClyA/GFP). Finally, the acid‐induced vectors were transformed into *E. coli* DH5*α* competent cells by an established heat shock method for recombination. After mass replication of plasmids, these were extracted and transformed into *E. coli* MG1655.

### Acid‐Induced ClyA Expression

*E. coli* MG1655 transformed with pET3a@adiA/ClyA/GFP (AIB@ClyA) were grown in LB solid medium (0.5 mg mL^−1^ NaCl, 10 mg mL^−1^ tryptone, 5 mg mL^−1^ yeast extract) overnight and transferred to a shaker‐incubator containing 100 µg mL^−1^ ampicillin. For the pH experiments, three different pH (5.0, 6.8, and 7.4) were used with the same amount of AIB@ClyA seed solution and incubated at 37 °C. At different incubation time points, a small amount of solution was taken out from the three shaker‐incubators and observed using a blue light instrument and fluorescence inverted microscope. The ClyA protein expression was analyzed from the fluorescence intensity of GFP. At the 12 h time period, the required amount of samples was taken from the three shaker‐incubators and centrifuged to obtain the supernatant, which was mixed with loading buffer (250 mm Tris‐HCl, pH 6.8, 0.5% BPB, 10% SDS, 50% glycerin, and 5% *β*‐mercaptoethanol), followed by incubation at 100 °C for 10 min. Finally, the mixture was resolved by sodium dodecyl sulfate‐polyacrylamide gel electrophoresis (SDS‐PAGE), His‐Tag mAb (1:5000), and anti‐mouse lgG (H+L) (1:10 000) were sequentially used for the Western blot assay.

### Cytotoxicity Assays In Vitro

CT26 cells were cultured in RPMI‐1640 medium containing 10% FBS and 1%‐1% penicillin‐streptomycin at 37 °C with 5% CO_2_. The relative cell viability of *E.coli* and AIB@ClyA was evaluated by MTT and transwell assays. Briefly, CT26 cells were seeded in the lower chamber of a 24‐well transwell and cultured overnight to cover the bottom section of the well. *E.coli* MG1655 and AIB@ClyA were incubated in shaker‐incubators overnight at various pH values. Next, 100 µL medium containing the same concentrations of *E.coli* and AIB@ClyA at various pH values were added into the upper chamber of the transwell. The transwell was cultured for another 24 h, and the upper chamber was removed and the medium was replaced with 60 µL MTT (5 mg mL^−1^) and incubated for another 4 h. The supernatant in each well was discarded and 500 µL DMSO was added. Cytotoxicity was determined by light absorption analysis at 570 nm according to the following formula:

(1) $$\begin{array}{ccc} \text{Relative}\;\text{cell}\;\text{viability}\;\left(\%\right) & = & \left({\mathrm{OD}}_{\mathrm{sample}}-{\mathrm{OD}}_{\mathrm{blank}}\right)/\\ & & \left({\mathrm{OD}}_{\mathrm{control}}-{\mathrm{OD}}_{\mathrm{blank}}\right)\times 100\% \end{array}$$

### Hemolysis Assay

Whole blood was taken from the heart of healthy Balb/c mice and stored in an anticoagulant tube, which was centrifuged at 5000 rpm for 5 min and the supernatant was discarded. The precipitated red blood cells were washed with PBS and redispersed in PBS. AIB@ClyA was incubated at pH 5.0 overnight and the culture medium supernatant was collected after centrifugation and freeze‐dried to obtain the protein for analysis. Red blood cells were incubated with various concentrations of the ClyA protein in PBS. Red blood cells incubated with deionized water and PBS were used as positive and negative controls, respectively. The mixtures were incubated at 37 ℃ for 8 h with shaking at 100 rpm. After incubation, these mixtures were centrifuged at 5000 rpm for 5 min for imaging analysis, and a microplate reader was used to measure the absorbance value of the supernatant at 570 nm. The percent hemolysis was calculated according to the following formula:

(2) $$\begin{array}{ccc} \mathrm{Hemolysis}\left(\%\right) & = & \left({\mathrm{OD}}_{\mathrm{sample}}-{\mathrm{OD}}_{\mathrm{negative}}\right)/\\ & & \left({\mathrm{OD}}_{\mathrm{positive}}-{\mathrm{OD}}_{\mathrm{negative}}\right)\times 100\% \end{array}$$

### Vasodilator Inflammatory Factor Detection

The serum levels of vasodilator inflammatory factors, including BK, LTC4, His, and PAF were detected using ELISA kits. Briefly, whole blood of CT26 tumor‐bearing mice was obtained from the heart at predetermined time points (0, 12, 24, and 36 h) after the intravenous injection of AIB@ClyA. After standing for 20 min, the whole blood samples were centrifuged at 2000 rpm for 20 min to collect the supernatant. The relevant tests were performed according to the manufacturer's instructions.

### Thrombosis Analysis

When the tumor volume reached ≈100 mm^3^, the mice were randomly divided into three groups (three mice per group) and treated with PBS, *E.coli* (10^8^ CFU per mouse), or AIB@ClyA (10^8^ CFU per mouse). At 24 h post‐injection, one mouse was sacrificed in each group to harvest the tumor tissues. The tumor tissues were sliced and stained with an anti‐CD31 antibody for analysis of blood vessels and an anti‐CD41 antibody for activated platelets analysis, using a fluorescence inverted microscope. Vascular leakage was studied by the additional injection of FITC‐dextran (20 mg mL^−1^, 150 µL). At 30 min after the injection, mice were sacrificed and tumor tissues were collected. The tumor tissues were sliced and stained with DAPI for analyzing the cell nucleus and observed using a fluorescence inverted microscope. The green area in the field of view was the dextran^+^ area, and the final intratumoral leakage area was estimated by calculating the green area as a percentage of the total area.

For tumor vascular perfusion analysis, Evans blue was intravenously injected 24 h after the different treatments. Three hours later, the mice were sacrificed and tumor tissues were harvested for photography. The tumors were soaked in formamide for 3 days to extract the Evans blue. The Evans blue concentration in tumors was quantified using a pre‐constructed standard curve by measuring the absorption at 620 nm with a UV–vis spectrophotometer.

### Nutrient Deprivation Analysis

Nutrient deprivation in tumor tissues was evaluated by metabonomic analysis. After treatment with AIB@ClyA for 24 h, the tumor tissues were collected and stored at −80 ℃, and tumor tissues collected from mice injected with PBS were used as controls. The samples for metabolomic analysis were prepared according to the operating instructions. Briefly, the extractant was prepared according to a 3:3:2 ratio of acetonitrile:isopropanol:water. Next, 10 mg of collected tumor tissues were mixed with 1 mL extractant, followed by ultrasound treatment on an ice bath for 1 min. Subsequently, the mixture was centrifuged at 1300 ×*g* for 5 min to collect the supernatant. Next, 1 mL of collected supernatant was mixed with 25 µL Myristic‐d27 acid to blow‐dried, followed by the addition of methoxyamine hydrochloride‐pyridine (25 µL) at room temperature. After 1.5 h, a 90 µL mixture of 1% trimethylchlorosilane and N‐methyl‐N‐(trimethylsilyl) trifluoroacetamide was added to the above mixture and coincubated at 37 °C for 30 min. Finally, the supernatant was collected by centrifugation and sent to Wuhan Metware Biotechnology Co., Ltd. for subsequent GC‐MS analysis.

### Animal Models

All animal experiments were performed according to the guidelines for laboratory animals established by the Wuhan University Center for Animal Experiment/A3‐Lab (W20170469). After a week of adaptive feeding, 7‐week‐old female BALB/c mice weighing ≈20 g received a subcutaneous injection of 10^6^ CT26 cells per mouse at the back on the right flank. When the tumor volume reached ≈100 mm^3^ (tumor volume = 1/2 × length × width^2^), the mice were used in the subsequent animal experiments.

### In Vivo Fluorescence Imaging and Tissue Distribution Analysis

For in vivo fluorescence imaging, DiR‐labeled *E.coli* and AIB@ClyA were prepared. Briefly, *E.coli* and AIB@ClyA were stained with DiR for 30 min, washed with PBS, and centrifuged to harvest cells for intravenous injection. When the tumor volume reached ≈100 mm^3^, the mice were intravenously injected with DiR‐labeled *E.coli* or AIB@ClyA (CFU≈10^8^, 100 µL). Thereafter, in vivo fluorescence imaging was performed on an IVIS Spectrum (PerkinElmer) platform at different time points. At defined time points, mice were sacrificed and tumors and organs (heart, liver, spleen, lung, and kidney) were harvested for fluorescence imaging.

### In Vivo Anti‐Tumor Study

When tumor volume reached ≈100 mm^3^, 15 mice were randomly divided into three groups. As a control, one group of mice was intravenously injected with PBS. The remaining two groups were injected with *E.coli* or AIB@ClyA via the tail vein with an equal dose of 10^8^ CFU per mouse. During the treatment, the body weight and tumor volumes of every mouse were monitored daily by electronic balance and caliper measurements, respectively. After the mice were mercifully sacrificed at 14 days post‐injection, the major organs and tumor tissues were harvested for immunofluorescence staining and histological analysis.

### In Vivo Safety Evaluation

To evaluate the physiological effects caused by AIB@ClyA, the tumor tissues and major organs of mice injected with AIB@ClyA were harvested at specific time points (0, 4, 12, 24, 48, and 96 h). The tumor tissues and major organs, including the heart, liver, spleen, lung, and kidney were extracted, weighed, and homogenized at 4 ℃ in sterile PBS. The samples were diluted and plated on LB agar plates. After overnight incubation, bacterial colonies were photographed and counted. Thereafter, the bacterial titer (CFU per gram of tissue) was calculated by colony counts and the tissue weight was analyzed to evaluate the retention of bacteria in the organ tissues in vivo. The blood samples of mice after injecting AIB@ClyA at different time points (0.5, 1, 3, and 7 days) were collected for blood biochemistry and blood routine analysis. Untreated mice were used as controls. After treatment, the main organs of mice in each group were also collected for H&E staining to analyze the lesions.

### Statistical Analysis

Statistical analysis was performed by using GraphPad Prism 8.0. Data were obtained from at least three independent measurements and were shown as the mean ± standard deviation (SD) unless otherwise indicated. Statistical analysis was performed using one‐way analysis or the unpaired two‐tailed Student's *t*‐test. *p*<0.01 and *p*<0.001 were considered to be statistically significant and indicated by ** and ***, respectively.

## Conflict of Interest

The authors declare no conflict of interest.

## Supporting information

Supporting Information

## Data Availability

The data that support the findings of this study are available from the corresponding author upon reasonable request.
