# Supplementary material for: Bacteria‐Elicited Specific Thrombosis Utilizing Acid‐Induced Cytolysin A Expression to Enable Potent Tumor Therapy
Source: Adv Sci (Weinh). 2022 Apr 11;9(15):2105086. doi: 10.1002/advs.202105086 (PMC9130894; doi:10.1002/advs.202105086)
Supplement: Supplementary file 1 — Supporting Information [file ADVS-9-2105086-s001.pdf]

ADVANCED SCIENCE
Open Access

Supporting Information

for *Adv. Sci.*, DOI 10.1002/advs.202105086

Bacteria-Elicited Specific Thrombosis Utilizing Acid-Induced Cytolysin A Expression to Enable Potent Tumor Therapy

Wenjun Qin, Wenxuan Xu, Longyu Wang, Debao Ren, Yibin Cheng, Wen Song, Tao Jiang, Lixin Ma and Cheng Zhang**

Supporting Information of

Bacteria-Elicited Specific Thrombosis Utilizing Acid-Induced Cytolysin A Expression to Enable Potent Tumor Therapy

Wenjun Qin, Wenxuan Xu, Longyu Wang, Debao Ren, Yibin Cheng, Wen Song, Tao Jiang, Lixin Ma*, and Cheng Zhang**

W. Qin

Ministry of Education Key Laboratory for the Green Preparation and Application of Functional Materials, School of Materials Science and Engineering, Hubei University, Wuhan 430062, P. R. China

E-mail: jiangtao@hubu.edu.cn

W. Xu, L. Wang, D. Ren, Dr. Y. Chen, Prof. L. Ma, Dr. C. Zhang

State Key Laboratory of Biocatalysis and Enzyme Engineering, Hubei Key Laboratory of Industrial Biotechnology, School of Life Sciences, Hubei University, Wuhan 430062, P. R. China

F-mail: malixing@hubu.edu.cn; zhangcheng1988@whu.edu.cn

Dr. W. Song

Institute of Biology and Medicine & College of Life Science and Health, Wuhan University of Science and Technology, Wuhan, 430081, P. R. China

Table S1. Primers used to construct pET3a@adiA/ClyA/GFP.

Name	Sequence (5’-3’)
adiA-F	GGCGTAGAGGATCGAGATCTGCCGCAACAATACCG
adiA-R	ATGATGATGATGGTGCATATGCATTGCTTACCCGGTTATGA
ClyA-F	ACCGGAATATTTGCAGAACAAACTGT
ClyA-R	TTAGCAGCCGGATCCTCAGACGTCAGGAACCTCGAAAAG
GFP-F	CATATGCACCATCATCATCATCATATGG
GFP-R	TGCAAATATTCCGGTTTTATACAGTTCATCCATGCCCAGATC

Table S2. Bacterial strains used in this study.

Bacterial strain	Plasmid	Use
E.coli DH5α	pET3a@adiA/ClyA/GFP	Construction vector
E.coli MG1655	pET3a@adiA/ClyA/GFP	Expression ClyA

Table S3. Reference value of blood biochemistry and blood routine indexes in mice.

Blood cells (10^9/L)			Liver function related enzymes (U/L)	
Lymph		3.6-11.56	ALT	40-170
WBC		5.69-14.84	GGT	<10
RBC		8160-11690	AST	67-381
	Eos	0.01-0.35	Kidney function related biomarkers (mM/L)	
Mid	Mon	0.34-1.37		
	Bas	0.00-0.16	UREA	2.50-11.07
PLT		476-1161	CRE	0.02-0.04
Gran		0.74-3.01	GLU	4.68-15.60

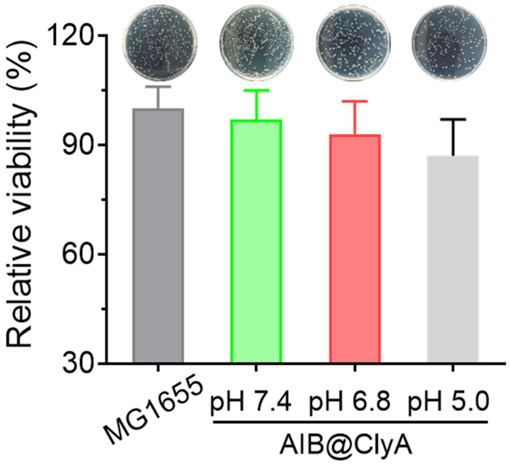

Figure S1. Relative viability of AIB@ClyA at various pH values.

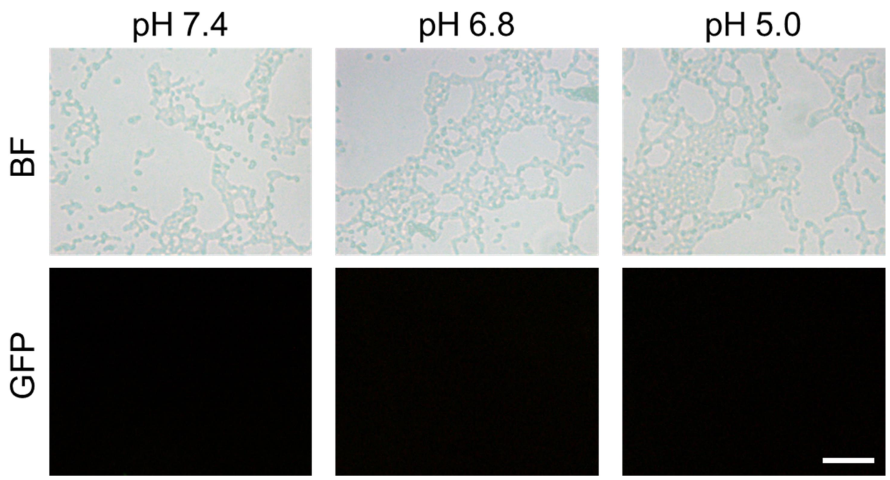

Figure S2. After culturing under different pH mediums for 16 h, the expression of GFP in *E.coli* MG1655 was observed by an inverted fluorescence microscope. Scale bar: 20 μm.

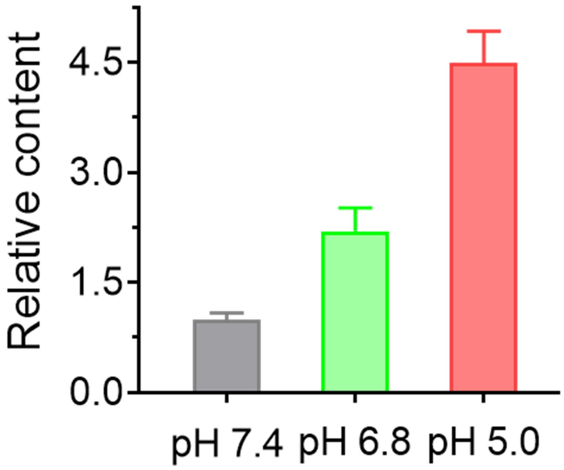

Figure S3. The relative content of ClyA expression by AIB@ClyA after 16 h of culture at various pH mediums.

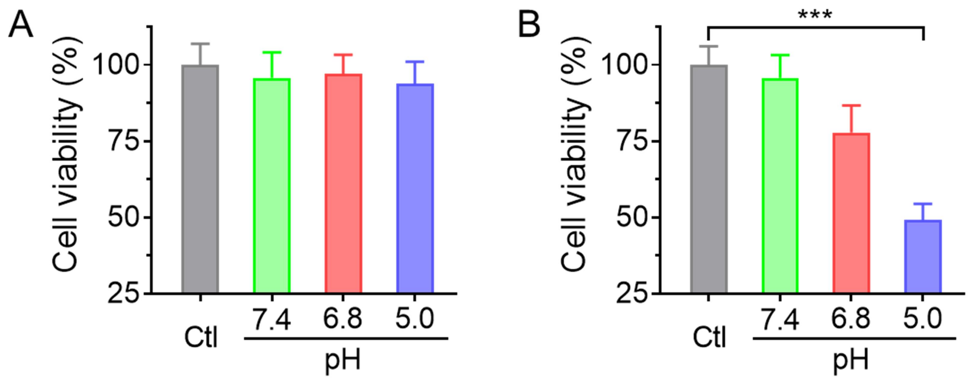

Figure S4. Cytotoxicity of A) *E.coli* MG1655 and B) AIB@ClyA after different pH mediums treated against CT26 cells in vitro. All data are presented as the means ± SD and statistical analyses were performed by unpaired two-tailed Student's t-test. ***$P < 0.001$.

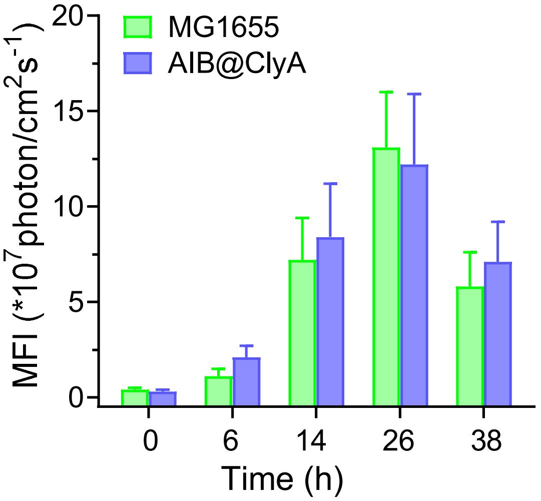

Figure S5. Mean fluorescence intensity (MFI) of tumor sites at different time points after injection of DiR labeled MG1655 and AIB@ClyA.

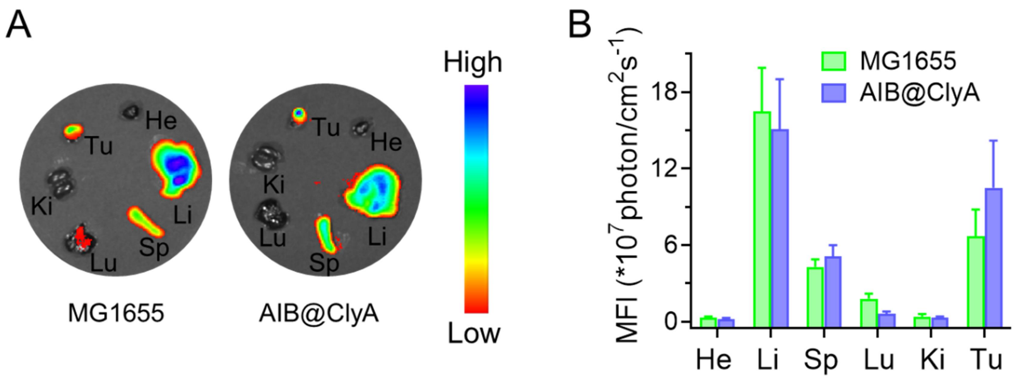

Figure S6. A) Fluorescence image of isolated organs and tumor tissues at 26 h post-injection (He-heart, Li-liver; Sp-spleen, Lu-lung, Ki-kidney, and Tu-tumor). B) Corresponding MFI of isolated organs and tumor tissues.

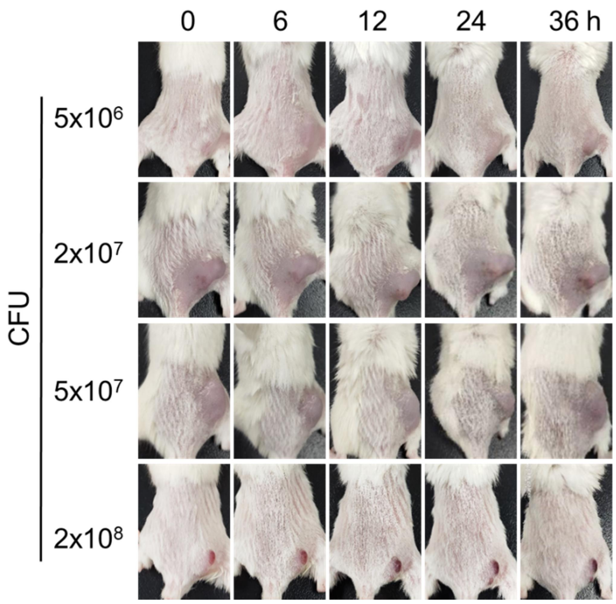

Figure S7. Photographs of CT26 tumor-bearing mice before or after injection of AIB@ClyA at different doses.

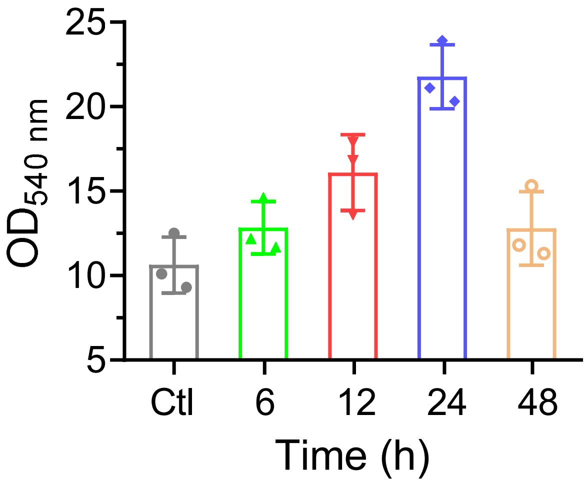

Figure S8. Hemoglobin content in tumor tissues at different time points after intravenous injection of AIB@ClyA.

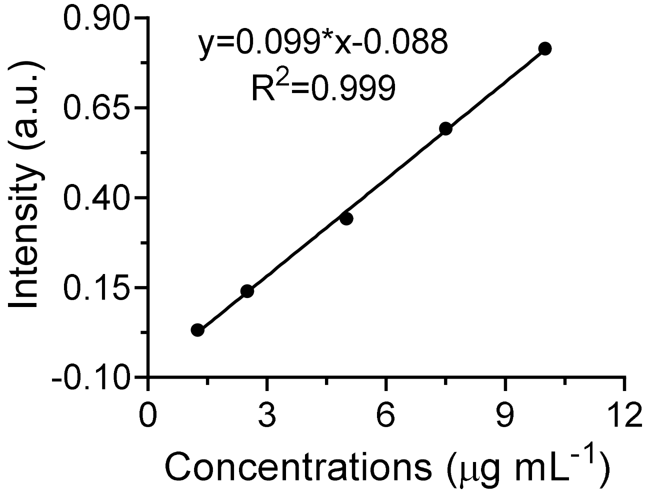

Figure S9. Linear relationships between the UV-vis absorbance and the concentration of Evans blue.

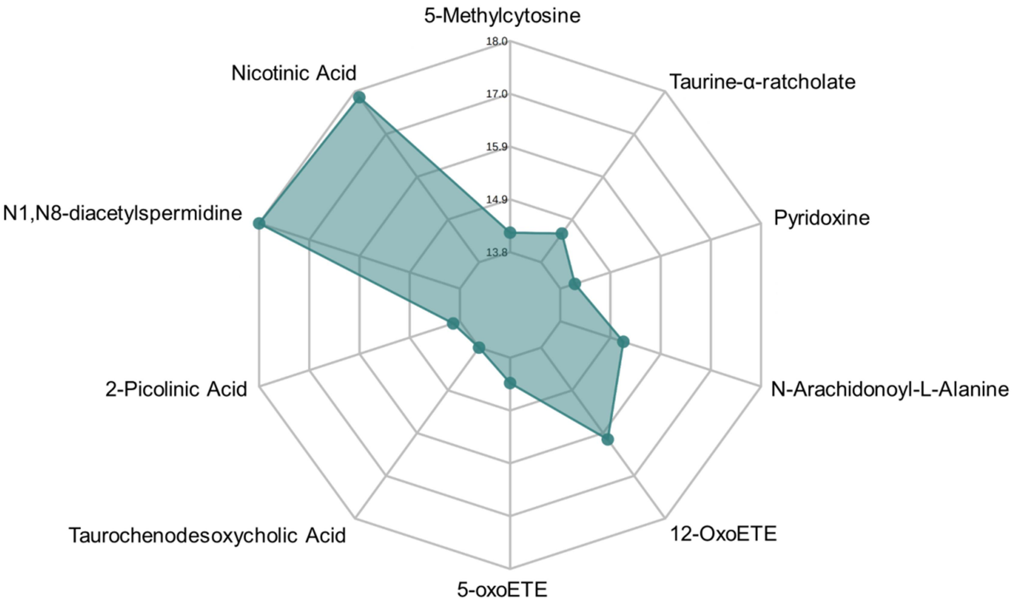

Figure S10. Radar map for the top 10 metabolites with the largest FC value. Gridlines represent log2FC values of differential metabolites.

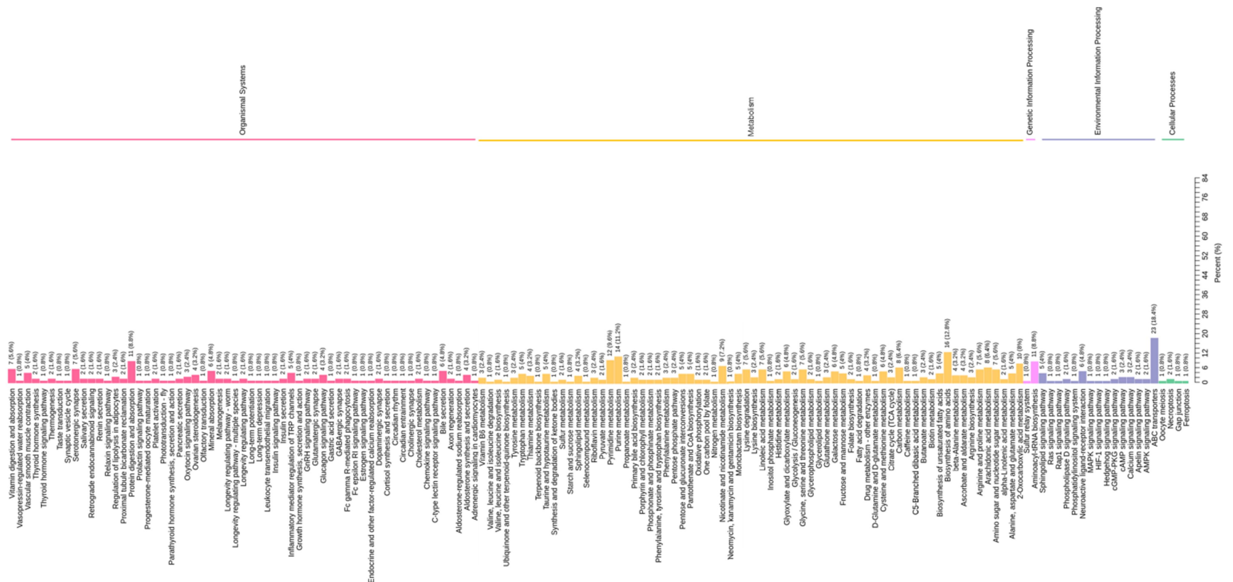

Figure S11. KEGG classification diagram of differential metabolites.

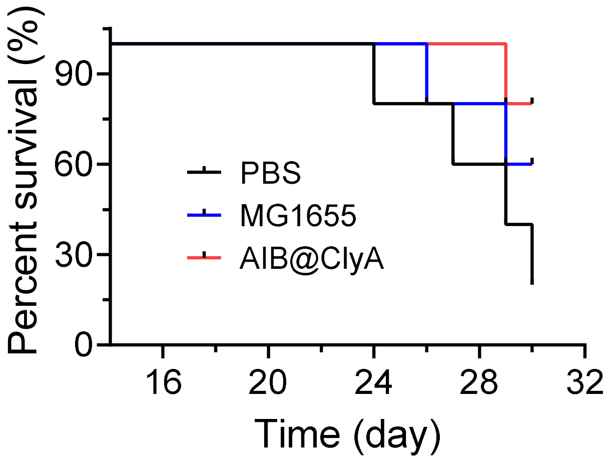

Figure S12. Survival curves of CT26 tumor bearing mice after different treatments on 30th day.

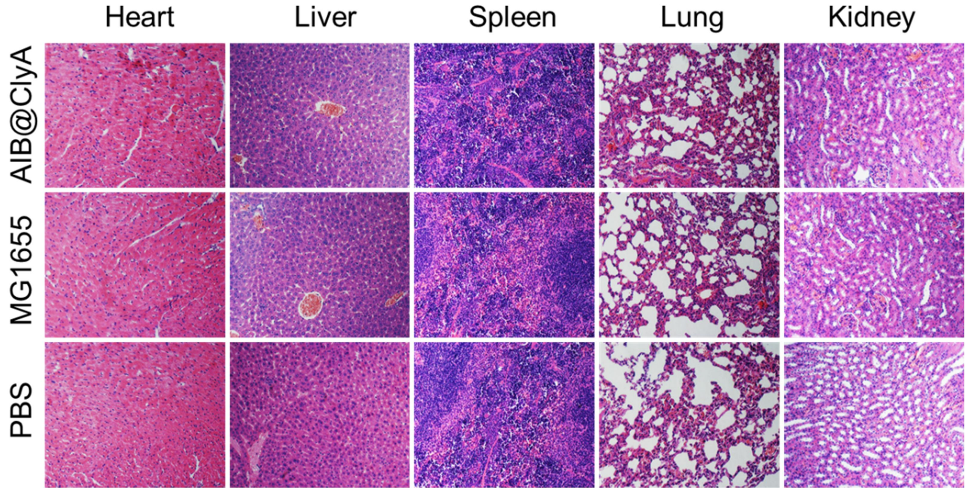

Figure S13. H&E staining of the major organs after different treatments.
